# How to Establish the Baseline for Non-Invasive Technological Regenerative Esthetic Medicine in the Face and Neck Region: A Literature Review

**DOI:** 10.3390/jpm15070283

**Published:** 2025-07-02

**Authors:** Ornella Rossi, Giovanna Perrotti, Riccardo Scaini, Massimo Del Fabbro, Giovanni Damiani, Tiziano Testori

**Affiliations:** 1 Department of Biomedical, Surgical, and Dental Sciences, University of Milan, 20122 Milan, Italy; riccardoscaini@me.com (R.S.); massimo.delfabbro@unimi.it (M.D.F.); giovanni.damiani1@unimi.it (G.D.); tiziano.testori@unimi.it (T.T.); 2 Lake Como Institute, 22100 Como, Italy; giovanna.perrotti@lakecomoinstitute.com; 3 Department of Implantology and Oral Rehabilitation, IRCCS Galeazzi-Sant’Ambrogio Hospital, 20157 Milan, Italy; 4 IRCCS Ca’ Granda Foundation, Ospedale Maggiore Policlinico, 20122 Milan, Italy; 5 Italian Center for Precision Medicine and Chronic Inflammation, University of Milan, 20122 Milan, Italy; 6 Department of Periodontics and Oral Medicine, University of Michigan School of Dentistry, Ann Arbor, MI 48109, USA; 7 Department of Oral Medicine, Infection and Immunity, Harvard School of Dental Medicine, Boston, MA 01451, USA

**Keywords:** high-intensity focused ultrasound, dynamic quadripolar diathermy, anti-aging, esthetic medicine, technological lifting, esthetic medicine baseline

## Abstract

(1) **Background**: Esthetic regenerative medicine is increasingly in demand for facial and neck rejuvenation due to its proven efficacy, safety profile, and minimal downtime. This study aimed to evaluate the role of standardized assessment tools in optimizing the outcomes of non-invasive regenerative esthetic technologies, both during the treatment course and in follow-up. (2) **Methods**: A literature review of the main articles published in peer-reviewed journals was conducted to identify high-quality studies addressing the use of validated esthetic scales and questionnaires (patient-reported outcomes) for evaluating the effectiveness of non-invasive regenerative treatments for the face and neck using accessible clinical tools such as photographs and 3D facial scanning. (3) **Results**: Clinician-reported outcomes (CROs) can be collected using standardized and reproducible photographic documentation and facial scans. The esthetic scales and classifications target both specific facial areas (e.g., upper third, perioral, periorbital) and overall skin appearance. Furthermore, advanced software allows overlay facial scan analysis and wrinkle mapping for precise quantification of improvements. In addition to objective CROs, patient-reported outcomes (PROs) offer essential insights into perceived esthetic changes, satisfaction, and emotional well-being, completing a multidimensional evaluation of treatment efficacy. (4) **Conclusions**: Standardized evaluation protocols based on accessible tools such as clinical photographs, 3D facial scans, and validated PRO questionnaires are essential for guiding effective, personalized regenerative treatments. Their integration into routine practice enhances clinical decision-making and patient satisfaction. While advanced tools like dermal probes may further refine assessments, they require specific expertise and resources and may be less practical for daily clinical use.

## 1. Introduction

The use of non-invasive technologies, such as ultrasound (US) [1,2,3,4,5,6], radiofrequency (RF) [7,8,9] and fractional laser (FL) [10], has become increasingly important in esthetic medicine for facial and neck rejuvenation, offering a safe and effective solution to enhance skin quality and promote tissue regeneration [10,11,12,13]. However, to ensure reliable and safe outcomes, these treatments must be performed with a thorough understanding of their mechanisms of action and associated contraindications, as well as an accurate assessment of their efficacy [12,13]. Non-invasive regenerative technologies are widely employed in esthetic medicine for their ability to stimulate natural biological processes and promote tissue rejuvenation, by heating targeted tissues at different depths to stimulate collagen and elastin production, enhancing elasticity and natural lifting effects and reducing visible signs of aging [1,2,3,4,5,7,8,9,10].

Regenerative esthetic medicine, by its nature, does not produce immediate results like injectable (filler, Botox) or surgical (facelift, blepharoplasty) esthetic techniques, which offer rapid and visible improvements. Instead, it relies on the stimulation of endogenous biological processes, promoting tissue regeneration over time [6,8]. While effects are not immediately apparent, the long-term benefits are significant: progressive improvements in skin tone, elasticity, and structure occur through a natural repair process at the cellular level [5]. This type of treatment, through its non-invasive approach, offers the advantage of achieving more harmonious and lasting skin aging, reducing the risks associated with invasive techniques, and stimulating natural skin regeneration mechanisms [13].

Given the gradual nature of these results, which depend on the continuous stimulation of biological processes, it is essential to monitor changes over time both clinically and through patient feedback. This approach allows the long-term benefits to be fully appreciated, maintaining patient confidence in the treatment and encouraging adherence to the therapeutic journey [6,10]. Establishing a precise baseline enables a clear and standardized evaluation of clinical variations in the short and long term, supporting clinical decision-making [5,12].

Understanding the precise mechanisms of action of different esthetic technologies not only enhances safety and efficacy but also facilitates the development of personalized evaluation methods [3,7]. With reliable tools and an accurate baseline, clinicians can monitor treatment progress, optimize esthetic results and meet patient expectations [4,9].

This study aims to identify the clinical and functional parameters necessary for an objective evaluation of esthetic treatment efficacy. It considers both skin texture and tissue elasticity, as well as patient feedback, to understand perceived improvements. To ensure objective and reproducible measurements, individual variations, such as age, skin type and health status, must be accounted for. Adopting standardized protocols to monitor changes over time aids in optimizing clinical decisions and effectively integrating esthetic medicine into clinical practice [10,11,12].

## 2. Study Objective

This study aims to provide clinicians with a guide for establishing a reliable and reproducible baseline to assess the short- and long-term efficacy of non-invasive regenerative medicine treatments for the facial area, minimizing confounding variables. The study intends to resume the most relevant rating scales in the literature and patient-reported outcome measures (PROMs) to define both objective and subjective clinical parameters, perceived by clinician and patient. This approach ensures a comprehensive evaluation of treatment results, accounting for individual factors such as age, skin type and general health status. Additionally, the study aims to establish a standardized assessment protocol that facilitate clinicians to monitor treatment induced changes over time.

## 3. Methods

A literature review was conducted to identify high-quality studies related to tools and scales to assess the outcome of non-invasive technological regenerative esthetic treatments for the face and neck, such as photographic evaluation, facial scanning, dermoscopy and patient-reported outcomes through questionnaires. Only articles in the English language published in peer-reviewed journals were included to ensure the selection of robust and reliable sources. No limitation was set regarding study design or publication year. The search was performed across major scientific databases, including PubMed, Scopus and Web of Science, using key words like esthetic medicine, skin regeneration, skin lifting, skin texture assessment, anti-aging, dynamic quadripolar diathermy and esthetic assessment protocol. Only studies considering evaluation scales and questionnaires, published in dermatological journals, plastic surgery journals, esthetic journals or cosmetic journals were included. As inclusion criteria, articles had to provide detailed methodologies, objective clinical assessments and patient-reported outcomes to ensure a comprehensive evaluation. Studies lacking rigorous methodological frameworks or published in non-indexed journals were excluded. No risk-of-bias assessment nor data extraction from included studies was performed due to the narrative nature of this review. The content of the articles was summarized in a descriptive way, according to the type of assessment of esthetics and skin features.

## 4. Results and Discussion

### 4.1. Facial Photographs

Facial photographs are essential tools for documenting and monitoring changes in the face–neck area from a clinical perspective, as Clinician Reported Outcomes (CROs). The use of high-quality photographs taken from standardized angles and lighting conditions ensures consistent and comparable visual documentation. Images can be used to compare pre- and post-treatment results, providing a tangible visual representation of improvements. Photographs taken from different perspectives allow both the clinician and the patient to observe changes clearly and objectively, facilitating an accurate assessment of treatment effectiveness, with a positive impact on the patient, who is motivated to continue the treatment.

Various scales are used in dermatology and esthetic medicine that allow the clinician to assess specific wrinkles or, more generally, the entire face using semi-quantitative scales. For specific assessment of upper-third wrinkles, the **Facial Wrinkle Scale (FWS)** provides a score for resting and maximum attempts at frowning, raising or smiling for the glabella, crow’s feet and forehead areas (Table 1) [14,15]. This rating scale is particularly used in the treatment of the upper third with botulinum toxin but can be used for regenerative medicine treatments specific to this area. For perioral and periorbital wrinkles, the **Fitzpatrick’s Classification of Facial Wrinkling** distinguishes between fine wrinkles with mild elastosis, moderately deep wrinkles with moderate elastosis and more numerous and deep wrinkles, with or without redundant skin and severe elastosis (Table 2) [16,17]. The **Wrinkle Assessment Scale (Lemperle G)** is a comprehensive classification that assesses the depth of wrinkles in 11 facial regions, assigning each zone a score from 1 to 6 (Table 3) [17]. However, these scales have limitations as they focus on specific areas of the face or particular depths and sizes of wrinkles, without considering the overall appearance of the face as a whole.

In contrast, other classifications focus on overall skin appearance and wrinkles and do not specifically address facial mimetic wrinkles. For instance, the **Glogau Classification** (Table 4) [18] evaluates chronological aging based on the presence and extent of wrinkles and skin color, assigning a ‘skin age’ and categorizing the patient into four categories (mild, intermediate, advanced and severe). The **Griffiths Photonumeric Scale** (Table 5) [19] is a standardized assessment scale that evaluates the degree of skin aging and visible signs of photoaging due to sun exposure. It is a photographic scale (using reference images to represent different aging or skin damage levels) and a numeric scale (each level is assigned a numerical score to quantify severity), providing an objective assessment based on visual observation of skin changes and including aspects like wrinkles, loss of elasticity, irregular pigmentation, telangiectasias and other typical signs of photoaging.

These recognized scales are essential tools for assessing the effectiveness of esthetic or dermatological treatments. They enable the documentation and monitoring of skin changes during the course of treatment, offering valuable guidance for personalized patient treatment. It is important to continue with such evaluations even months and years after treatment completion, as regenerative medicine treatments often produce gradual effects and long-term improvements [5,6,8]. This distinguishes them from injectable treatments, whose results tend to fade within 6–18 months of injection. Therefore, assessments should be made before, during and at the end of treatment, as well as in the months following, to detect any delayed benefits.

### 4.2. Facial Scanning

Facial scanning is an advanced technology that provides a three-dimensional representation of the face, allowing precise analysis of facial structure and characteristics, such as symmetry, skin texture and tissue volume. This technology enables objective monitoring of changes over time, providing valuable data to accurately assess treatment efficacy [20,21]. In particular, facial scanning is valuable for quantifying improvements in skin tone and wrinkle reduction, contributing to a comprehensive full-face assessment of results [22]. This technology can be used to analyze chronological and photoaging through the same assessment scales employed for photographs [23]. Moreover, specific software allows for overlaying facial scans, using fixed and constant landmarks to ensure precision in comparative analysis [24]. An additional development is wrinkle mapping, which measures the depth of skin folds [23,25]. Advanced scanners designed for skin analysis collect detailed data on skin topography (wrinkles, texture and pigmentation) and produce high-resolution 3D images. These scanners use dedicated software to generate quantitative data and colored maps (e.g., wrinkle depth maps), ensuring constant lighting conditions and minimizing environmental interference. The system allows the visualization and measurement of skin surface elevations, depressions and variations in 3D. However, measurements can be influenced by factors such as applied pressure or patient position changes: standardization of the protocol, which requires a learning curve, minimizes such effects [24].

### 4.3. Patient-Reported Outcome (PROs)

In addition to objective clinical outcomes (CROs), it is essential to consider patient-reported outcomes (PROs), which are subjective measurements that reflect the direct experience of patients through questionnaires. These data reflect the patient’s perspective, their perception of esthetic changes, satisfaction with the treatment, wrinkle reduction and improvements in self-esteem. Patient feedback is crucial for fully understanding treatment effectiveness. Integrating PROs with CROs provides a more comprehensive evaluation, which not only measures objective physical improvements but also considers the psychological well-being and satisfaction of the patient.

Among the most widely used questionnaires in esthetic medicine is the **Facial Aesthetic Clinical Evaluation-Questionnaire (FACE-Q)** [26,27,28], a comprehensive tool divided into three main sections (Table 6). The first part assesses the esthetic perception of the face, including aspects such as symmetry, proportions, freshness, resting appearance and the profile in specific situations (e.g., in photos or under bright lighting). Patients express their satisfaction using a Likert scale from “very dissatisfied” to “very satisfied.” The second section evaluates psychological well-being, exploring aspects such as self-acceptance, confidence, feeling attractive and positive attitudes toward one’s image, using a scale from “strongly disagree” to “strongly agree.” The final part focuses on perceived age, asking patients to indicate on a **Visual Analog Scale (VAS)** how much younger or older they feel compared to their actual age, with scores ranging from −15 (looking 15 years younger) to +15 (looking 15 years older).

The **Facial Line Outcome (FLO-11)**, an extended version of the previous FLO-7 with only seven questions [29], is a questionnaire that evaluates the effectiveness and satisfaction of esthetic treatments for wrinkles, using a scale from 0 to 10. It examines the patient’s expectations (appearance improvement, treatment duration and naturalness), the emotional impact of wrinkles (e.g., appearing tired or angry) and satisfaction with the results. It also includes the probability of continuing or recommending the treatment and measures how well initial expectations were met. It is a valuable tool for assessing esthetic effects, psychological well-being and patient satisfaction with the treatment (Table 7) [30,31].

The **Face-Lift Surgery Questionnaire (FLS-Q)** is a tool primarily used to evaluate the results of facial lifting surgeries, but it is also applicable in regenerative esthetic medicine. It analyses esthetic satisfaction, psychological impact and the patient’s quality of life at baseline and post treatment during follow-up. The questionnaire helps monitor the patient’s perception and recovery experience, making it particularly useful for optimizing and evaluating treatment effectiveness (Table 8) [32].

**Figure 1:**
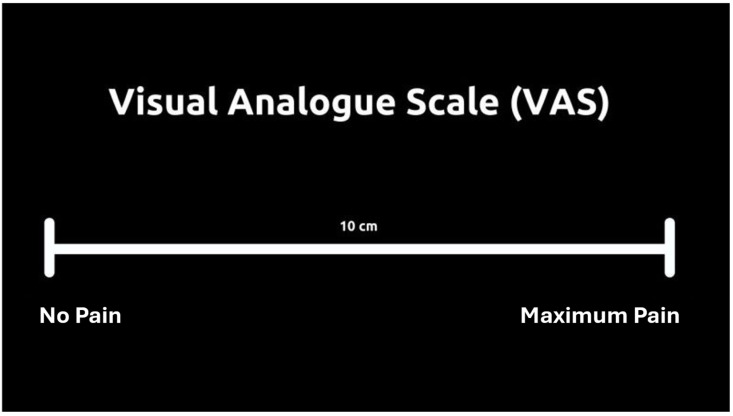
Pain—VAS: pain evaluation during treatment. Have the patient mark a point on the line; score: 0 to 4 mm—“no pain”; 5 to 44 mm—“mild pain”; 45 to 74 mm—“moderate pain”; and 75 to 100 mm—“severe pain”.

To complement this evaluation, the **Pain Visual Analog Scale (Pain-VAS)** is used to measure pain intensity, providing a clear and objective indication of the discomfort felt by the patient during and after treatment, with the goal of monitoring recovery progress (Figure 1) [33,34].

The **Self-Perception of Age (SPA)** allows the comparison of perceived age with actual age, asking patients to indicate whether they believe they appear their age, younger or older, specifying the number of years of difference. Patients also assess their perceived age during the seven days prior, providing a useful measure for esthetic treatments related to rejuvenation [35,36].

To assess patient satisfaction regarding the treatment of facial lines, **the Facial Line Treatment Satisfaction (FTS) Questionnaire** is used, which measures the level of satisfaction of patients after an esthetic or regenerative facial treatment. It consists of 14 statements addressing aspects such as overall satisfaction, improvement of facial lines, appearance, self-confidence and absence of side effects or visible treatment signs. Patients rate their satisfaction on a seven-point scale, ranging from “very dissatisfied” to “very satisfied.” This standardized questionnaire allows consistent evaluations of treatment outcomes and helps guide adjustments to improve patient satisfaction (Table 9) [37,38].

The combined use of clinician-reported outcomes (CROs), patient-reported outcomes (PROs) and instrumental evaluations such as facial photographs and 3D facial scanning offers a multidimensional approach to the assessment of non-invasive technological regenerative esthetic treatments. Facial photographs provide visual documentation and enable comparative assessments using standardized scales to objectify wrinkle depth, photoaging and dermal quality. Facial scanning enhances this evaluation with high-resolution, three-dimensional analyses of skin texture, volume and symmetry, allowing precise quantification of structural changes. Complementing these objective measures, validated patient questionnaires capture the subjective dimension of treatment effectiveness, including satisfaction, perceived improvement, psychological well-being and quality of life. When integrated, these tools allow a more comprehensive and personalized evaluation framework that reflects both the clinical efficacy and patient-centered outcomes of esthetic procedures. This holistic approach improves treatment planning, supports long-term monitoring and contributes to evidence-based practice in esthetic regenerative medicine.

## 5. Limitations and Future Directions

A limitation of the current evaluation approach lies in the reliance on questionnaires, photographs and facial scans, which predominantly yield qualitative data based on the clinician’s subjective assessment, rather than quantitative metrics suitable for pre- and post-treatment comparison. For even more thorough and precise evaluation, the integration of additional tools, such as dermal probes (cutometer, impedance meter, friction meter, corneometer, sebometer, etc.) assessing the physical and biochemical properties of the skin, represents an important step in closely monitoring skin structures and subcutaneous changes. These devices, combined with advanced ultrasound imaging techniques, could provide a more comprehensive view of skin health and treatment response, further improving the personalization of esthetic and anti-aging treatments. However, these tools are often more expensive, specific to esthetic treatment and cosmetology and require considerable expertise for correct use and interpretation of the results. Unlike photographs or facial scanners, which are more commonly accessible and easier to use in clinical practice, dermal probes may be less practical for routine use by professionals

## 6. Conclusions

Esthetic regenerative medicine technologies are particularly valuable for their high efficacy, safety and short recovery time, characteristics that make them ideal for patients seeking esthetic improvements without invasive surgery. Moreover, anti-aging treatments not only improve esthetic appearance but also contribute to skin health by preventing skin diseases and stimulating cellular regeneration.

The use of systematic evaluation tools, such as facial photography, facial scans and patient-specific questionnaires, is crucial for monitoring and optimizing the effectiveness of non-invasive esthetic treatments in regenerative medicine. These tools allow clinicians to establish accurate and reproducible baselines, track changes over time and tailor treatments to each patient’s unique needs and expectations. Adopting such systematic approaches not only enhances the personalization of care but also promotes long-lasting, safe and highly effective results, providing increasingly targeted solutions to meet the individual needs of patients.

For this reason, the use of aging and photoaging evaluation scales and a precise documentation protocol remain essential elements for optimizing treatments and ensuring their long-term effectiveness.

This approach supports clinical decisions based on solid data, facilitating the effective integration of non-invasive esthetic medicine into daily clinical practice, considering both objective measurements such as skin texture and elasticity and subjective feedback from patients and clinicians.

## Figures and Tables

**Table 1:** FWS: wrinkle scale for the upper face at baseline. Assign a score at rest and during maximum effort of frowning, elevation or forced smile: 0 = none; 1 = mild; 2 = moderate; and 3 = severe.

ITEM		SCORE	
		Baseline	Follow-Up
1	Glabella repose		
2	Glabella at maximum contraction		
3	Crow’s feet repose (bilaterally symmetrical)		
4	Crow’s feet at maximum contraction (bilaterally symmetrical)		
5	Forehead repose		
6	Forehead at maximum contraction		

**Table 2:** Fitzpatrick’s Classification of Facial Wrinkling. Evaluation of perioral and periorbital wrinkles. Assign a score of 1–9, classified as follows: Class I (score 1–3) MILD: fine wrinkles. Minor texture alterations with subtly accentuated skin lines. Class II (score 4–6) MODERATE: fine-to-moderate depth wrinkles with moderate number of lines. Distinct popular elastosis, individual papules with yellow translucency and dyschromia. Class III (score 7–9) SEVERE: fine-to-deep wrinkles, numerous lines with or without redundant skin. Multipapular and confluent elastosis, thickened yellow and pallid cutis rhomboidalis.

ITEM	WRINKLES	DEGREE OF ELASTOSIS	SCORE
1	Perioral		
2	Periorbital		

**Table 3:** Wrinkle Assessment Scale (Lemperle G). Evaluation of wrinkle depth in 11 facial regions. Assign a score from 0 to 6: 0 = no wrinkles; 1 = just perceptible wrinkles; 2 = shallow wrinkles; 3 = moderately deep wrinkles; 4 = deep wrinkles with well-defined edges; 5 = very deep wrinkles; 6 = redundant folds.

ITEM	TYPE OF WRINKLES	SCORE
1	Horizontal forehead lines	
2	Glabellar frown lines	
3	Periorbital lines	
4	Preauricular lines	
5	Cheek folds	
6	Nasolabial folds	
7	Upper lip lines	
8	Corner of mouth lines	
9	Marionette lines	
10	Chin Crease	
11	Neck folds	
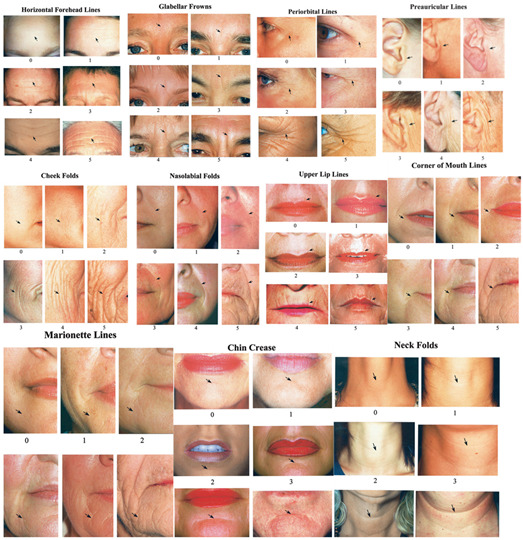		

**Table 4:** Glogau Classification. Chrono-aging assessment based on wrinkle presence and severity. Assign a score of 1–9: Type I (28–35 years), MILD (no wrinkles). Early photoaging, mild pigmentary changes, no keratoses and minimal wrinkles. Patient age: twenties or thirties. Minimal or no makeup (score 0). Type II (35–50 years), MODERATE (wrinkles in motion). Early-to-moderate photoaging, early senile lentigines visible, keratoses palpable but not visible and parallel smile lines beginning to appear. Patient age: late thirties or forties. Usually wears some foundation (score 1–3). Type III (50–65 years), ADVANCED (wrinkles at rest). Advanced photoaging, obvious dyschromia, telangiectasia, visible keratoses and wrinkles even when not moving. Patient age: fifties or older. Always wears heavy foundation (score 4–6). Type IV (65–80 years), SEVERE (only wrinkles). Severe photoaging, yellow-gray color of skin, prior skin malignancies, wrinkled throughout and no normal skin. Patient age: sixties or seventies. Cannot wear makeup, “cakes and cracks”(score 7–9).

ITEM	AGE	GRADE	SCORE
1			

**Table 5:** Griffiths Photonumeric Scale. Assessment of photodamage and repair after treatment. Assign a score of 0–9: 0 = none, 1–3 = mild, 4–6 = moderate and 7–9 = severe.

ITEM			SCORE
1	Fine wrinkles	This factor represents a visual assessment of the number and depth of superficial wrinkles (i.e., shallow indentations or lines). Fine wrinkles typically appear in periorbital and perioral regions and are usually found further from the eyes and mouth than are coarse wrinkles.	
2	Coarse wrinkles	This factor represents a visual assessment of the number and depth of coarse wrinkles (i.e., deep lines, furrows or creases). Coarse wrinkles appear on the forehead, glabella, chin and nasolabial and periorbital areas, and they tend to be located closer to the eyes and mouth than fine wrinkles.	
3	Mottled hyperpigmentation	This factor represents a visual assessment of light, patchy, mottled hyperpigmentation and solar freckling (including melasma) based on quantitative and qualitative criteria such as the area/density of pigment, color intensity (dark vs. light) and uniformity of distribution (i.e., the more uneven or blotchy, the greater the score). Lentigines, nevi and other pigmented lesions are not to be included in this assessment.	
4	Yellowing (sallow complexion)	This factor represents a visual assessment of color tones from very pink or rosy to very sallow or pale. Each parameter is assessed, and the overall severity is rated as follows	

**Table 6:** FACE-Q: facial evaluation, psychological well-being and aging. Rate from 1 to 4 (1 = very dissatisfied/strongly disagree, 2 = slightly dissatisfied/slightly disagree, 3 = quite satisfied/agree and 4 = very satisfied/strongly agree) and rate from −15 to +15.

SCALE	EVALUATION	MEASUREMENT OF RESPONSES
Facial Evaluation Scale	Patients answered the following questions to assess satisfaction: (1) How symmetrical is your face? (2) How balanced is your face? (3) How well-proportioned is your face? (4) How does your face look at the end of the day? (5) How fresh does your face look? (6) How rested does your face look? (7) How does your profile (side view) appear? (8) How does your face look in photos? (9) How does your face look when you first wake up? (10) How does your face look under bright lights?	1 = Very dissatisfied 2 = Slightly dissatisfied 3 = Quite satisfied 4 = Very satisfied
Psychological Well-being Scale	Patients indicated their agreement with the following statements: (1) I feel okay with myself (2) I am accepting myself (3) I am comfortable with myself (4) I feel good about myself (5) I like myself (6) I feel positive about myself (7) I feel happy (8) I feel attractive (9) I feel confident (10) I feel great about myself	Possible responses: 1 = Strongly disagree 2 = Slightly disagree 3 = Quite agree 4 = Strongly agree
Assessment of Aging Appearance	Patients answered the following question: “How many years younger or older do you think you look compared to your actual age?”	Patients circled a number on a VAS ranging from: −15 = I look 15 years younger 0 = I show my age +15 = I look 15 years older

**Table 7:** FLO-11: rate your wrinkles—0 = not at all, 5 = somewhat and 10 = very much.

ITEM	CONCEPT
1	My wrinkles make me feel less attractive
2	I feel less attractive compared to how others want me to appear
3	They make me look stressed
4	I feel comfortable with my wrinkles
5	I am bothered by my wrinkles
6	They make me feel tired
7	I look older than my age
8	I look angry
9	My wrinkles make me feel older than others think I appear
10	They don’t make me look well-rested
11	My skin isn’t smooth enough

**Table 8:** FLS-Q: evaluation of esthetic satisfaction, psychological impact and quality of life of the patient at baseline and post treatment (follow-up). Rate each item from 1 to 10.

DOMAIN	FLS-Q-Baseline
Treatment Expectations	Improvement of facial appearance Treatment initiation Treatment duration Achieving a natural appearance Overall treatment satisfaction Improvement of self-esteem
Impact	7. Feeling older 8. Negative impact on self-esteem 9. Looking tired 10. Feeling unhappy about facial wrinkles 11. Looking angry
**DOMAIN**	**FLS-Q-Follow-Up**
Treatment Satisfaction	Improvement of facial appearance Treatment initiation Treatment duration Achieving a natural appearance Treatment effect on wrinkles
Impact	6. Feeling older 7. Negative impact on self-esteem 8. Looking tired 9. Feeling unhappy 10. Looking angry
Continue the Treatment	Probability of continuing the treatment
Recommend the Treatment	2. Probability of recommending the treatment to a friend
Expectations Met	3. Extent to which treatment expectations were met

**Table 9:** FTS: treatment satisfaction (7-point scale: 1 = very dissatisfied, 2 = dissatisfied, 3 = slightly dissatisfied, 4 = neutral (neither satisfied nor dissatisfied), 5 = quite satisfied, 6 = satisfied and 7 = very satisfied) and importance evaluation (5-point scale: 1 = not important, 2 = slightly important, 3 = important, 4 = very important and 5 = extremely important).

ITEM		Satisfaction	Importance
**Treatment Effects**			
1	Overall satisfaction		
2	Improvement of facial wrinkles		
3	Time to onset of effects		
4	Improvement in appearance		
5	Looking rested		
6	Appearing rested		
7	Looking better		
8	Looking younger		
9	Looking how you feel		
10	Confidence		
11	Competitive at work		
**Procedure**			
12	Side effects		
13	No signs of the procedure		
14	No downtime		

## Data Availability

No new data were created or analyzed in this study. Data sharing is not applicable to this article.
